# Improving tuberculosis case detection in underdeveloped multi-ethnic regions with high disease burden: a case study of integrated control program in China

**DOI:** 10.1186/s40249-017-0365-4

**Published:** 2017-11-29

**Authors:** Jun Li, Xiao-Qiu Liu, Shi-Wen Jiang, Xue Li, Fei Yu, Yan Wang, Yong Peng, Xiao-Ming Gu, Yan-Ni Sun, Hui Zhang, Li-Xia Wang

**Affiliations:** 1 Jockey School of Public Health and Primary Care, Chinese University of Hong Kong, Hong Kong, China; 2 National Center for TB Control and Prevention, China CDC, 0515, 155 Changbai Road, Changping District, Beijing, 102206 China; 3 Yining Center for Disease Control and Prevention, Yili, China; 4 Yili Center for Disease Control and Prevention, Yili, China; 5 Xinjiang Center for Disease Control and Prevention, Urumqi, China; 6 World Health Organization Beijing Office, Beijing, China

**Keywords:** Tuberculosis, TB control program, Outreach education, Household screening, Case study/pilot, Ethnic groups, Aged/older people, Xinjiang/China

## Abstract

**Background:**

In the underdeveloped multi-ethnic regions of China, high tuberculosis (TB) burden and regional inequity in access to healthcare service increase the challenge of achieving the End TB goals. Among all the provinces, the highest TB burden is reported in Xinjiang, where ethnic minorities and older people have suffered most. However, current case-finding strategy is inadequate given the complex social determinants and suboptimal case detection rates. Thus, we developed an integrated TB control program to improve case detection and conducted a pilot in Xinjiang from 2014 to 2015. In this case study, we summarized the activities and key findings. We also shared the experiences and challenges of implementing interventions and provided recommendations to inform the TB control program in the future.

**Case presentation:**

The pilot interventions were implemented in one selected town in Yining based on local TB control programs. By applying tailor-made educational materials, outreach TB educational activities were conducted in diverse ways. In 22 Masjids, the trained imams promoted TB education to the Muslims, covering 20,440 person-times in 88 delivered preaching sessions. In seven schools, 1944 students were educated by the teachers and contributed to educating 6929 family members. In the village communities, 13,073 residents participated in household education and screening. Among them, 12,292 people aged under 65 years were investigated for suspicious pulmonary TB symptoms, where six TB patients were diagnosed out of 89 TB suspects; 781 older people were mobilized for screening directly by chest X-ray, where 10 patients were diagnosed out of 692 participants. Supportive healthcare system, multi-sectoral cooperation and multi-channel financing mechanism were the successful experiences of implementation. The interventions were proved to be more effective than the previous performance: the number of TB suspects consulting doctors and patients detected increased by 50% and 26%, respectively. The potential challenges, implications and recommendations should been taken into account for further program improvement.

**Conclusions:**

In underdeveloped multi-ethnic regions with high TB burden, improving case detection is necessary and the interventions can be feasible and effective within a supportive system. More intensive educational and training approaches, a high index of TB suspicion and prioritization of older people in screening are recommended. To sustain and scale up the program, the impacts, cost-effectiveness, feasibility and acceptability of interventions warrant further research and evaluation in each specific context.

**Electronic supplementary material:**

The online version of this article (10.1186/s40249-017-0365-4) contains supplementary material, which is available to authorized users.

## Multilingual abstracts

Please see Additional file 1 for translations of the abstract into the five official working languages of the United Nations.

## Background

Tuberculosis (TB) is one of the world’s deadliest infectious diseases of poverty. Every year, it causes vast majority of morbidity and mortality in the low and middle income countries [1]. Since 2015, the World Health Organization (WHO) reported TB ranked alongside human immunodeficiency virus as the leading cause of death from infectious disease [1]. In China, although remarkable achievement had been made by directly observed treatment, short-course (DOTS) strategy since the 1990s, it still has the world’s third largest TB burden. Regional inequity of TB epidemic distribution and in access to healthcare service also poses a huge challenge [1, 2]. According to China’s Fifth National TB Epidemiological Survey in 2010, the prevalence of bacteriologically confirmed pulmonary TB in the western region was 1.7 times and 3.2 times that of the central and eastern region, respectively [3]. Potential social determinants, including the low socioeconomic position and high proportion of ethnic minority groups, affected the progress of TB control and become a major barrier for disease elimination [4].

Xinjiang Uygur Autonomous Region (Xinjiang) is the largest provincial administrative division, which accounts for one-sixth of the nation’s territorial area [5]. It is located in north-western China and the heart of the ancient Silk Road. The current Xinjiang residents are composed of 47 China’s officially recognized ethnic groups, among whom the Uygur accounts for majority of residents [5]. The gross domestic product (GDP) per capita in Xinjiang was 6664 US dollars in 2014, below the national average level (7644 US dollars) [6, 7].

Despite the wide coverage of DOTS strategy in Xinjiang since 2000, consistently high TB burden poses great threat to public health and socioeconomic development. In the last decade, its TB notification rate was highest among all provinces in China, and continuously ranked within top two among all notifiable infectious diseases in Xinjiang [8]. The prevalence of bacteriologically confirmed pulmonary TB in Xinjiang increased from 365/100000 in 2000 to 430/100000 in 2011, which was much higher than the prevalence in the whole nation (116/100000) and western region (212/100000) [3, 4]. The uneven distribution of TB burden was also demonstrated within the region in various ethnicity and age groups. The TB prevalence in Uygur and other ethnic minority groups was respectively 6.9 and 4.5 times that of Han nationality; the prevalence was five times higher in older people (aged 65 years and over) than younger people (between 15 and 24 years old) [4].

In order to accelerate TB elimination, the WHO End TB Strategy outlines targets at 90% reduction in TB incidence and 95% in TB mortality by 2035 [9]. Achieving the targets is challenging in China, especially in those underdeveloped multi-ethnic regions like Xinjiang. In addition to the high TB burden, underdiagnosis or a longer delay in diagnosis and treatment would result in higher risk of new transmission and unfavourable treatment outcomes [10]. It was evaluated that more than half of TB patients with suspicious pulmonary TB symptoms (hereafter termed “symptoms”) in China didn’t consult any doctors mainly because of poor TB awareness and financial difficulty [11]. In a big-scale study, the median time of patient delay in diagnosis was 93 days (range 68 – 128), and even longer among older people, rural residents and people with limited access to health services [12]. According to the Xinjiang survey in 2011, the patient diagnosis rate was only 34% and it was lower in the Uygur (31%) [4]. The public awareness of TB knowledge was 48%, below the national level (57%) [11, 13]. Other factors such as language barriers, lack of health information, and living too far from medical institutions may also affect or delay the TB detection [14].

More efforts should be made given the high TB burden and suboptimal case detection performance. Although the WHO recommends to optimize actions along patient-initiated pathway and conduct systematic screening in specific high-risk groups for TB, little experience of targeted interventions or programs had been found in such settings [15, 16]. Therefore, we developed an integrated TB control program to improve case detection and conducted a pilot in Xinjiang from 2014 to 2015. In this case study, we summarized the activities and key findings. We also shared the experiences and challenges of implementing interventions and provided recommendations to inform the TB control program in the future.

## Case presentation

### Pilot setting

**Fig. 1:**
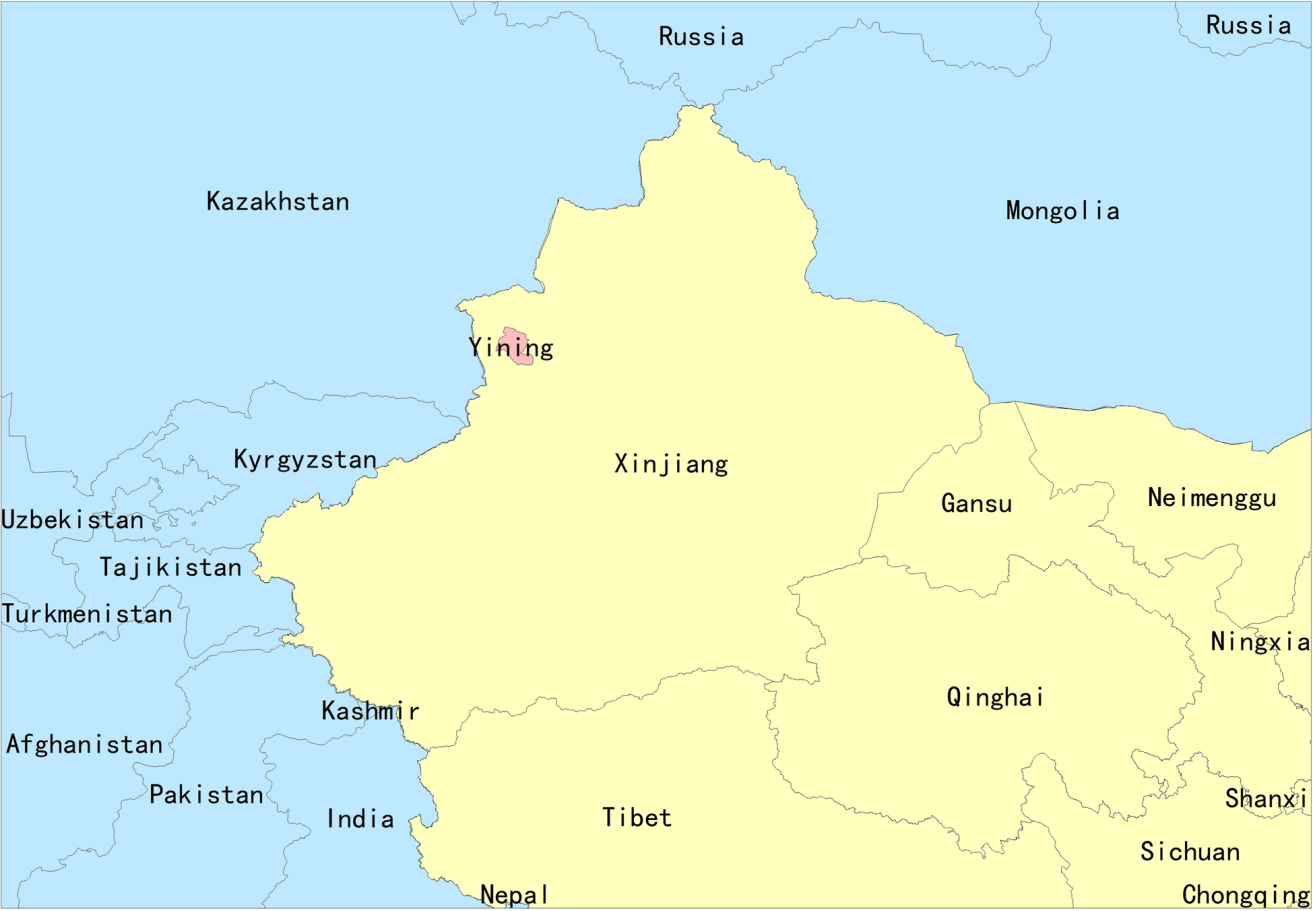
Map of pilot site where interventions were implemented

In the process of selecting pilot site in Xinjiang, we comprehensively considered the demographic structure of multi-ethnic residents, healthcare system and infrastructure, capability and willingness of implementation, as well as the representativeness of TB problems to be addressed. Accordingly, we selected one town in Yining County of Yili Prefecture that locates in the west of Xinjiang (Fig. 1). In Yining, more than 30 minority nationalities account for 84% of the populations (the Uygur accounts for 47%) with the remainder of Han [6]. The annual GDP per capita is around 1800 US dollars on average [6]. Yining has a well-established healthcare system, primary health insurance and control program for TB. Joint accountability and responsibility are stated and monitored by the Bureau of Health: TB designated hospital (hereafter termed “TB hospital”) is responsible for clinical diagnosis, treatment and management of TB patients; township health centres and village clinics conduct referral and trace of TB suspects (those with symptoms and/or abnormal chest X-ray presentation) and patients, close contact screening, health education and directly observed treatment. Centre for disease control and prevention (CDC) is in charge of TB program management, surveillance, health promotion, training, monitoring and evaluation. The new rural cooperative medical scheme that covered more than 95% of residents in Yining has allowed reimbursing 100% for TB outpatient and 90% for TB hospitalization since 2013 [17].

### Organization, funding and implementation

Under the leadership of national and regional Commissions of Health and Family Planning, China CDC was responsible for program development, and pilot design, training, monitoring, evaluation and management in cooperation with regional CDCs. In Yining, a leadership and implementing management team (consisted of the governments, departments of health, education, ethnic and religious affairs) took charged of pilot organization, coordination, enhanced training and implementation. The Xinjiang governments provided specific TB funds to implement interventions on the basis of national and local TB control programs. Technical supports were sponsored by China-WHO Biennial Collaborative Projects 2014-2015.

From March to August 2014, the pilot site selection, protocol and detailed implementation rules were finalized in line with field investigations, multi-party discussions and pre-test. The opinions from representatives of imams, school teachers, and healthcare workers were integrated. Pilot milestone targets were set for the purpose of quality control. In September 2014, a comprehensive launching and training conference was held in Yining with participations from all related facilities. Separate enhanced training courses were held in each homogeneous group to specify their responsibilities and rules in activities. From October to December 2014, the pilot was implemented under technical guidance and monitoring by national and regional TB experts. TB patients were initially diagnosed by clinicians at the TB hospital and further confirmed by national TB clinical experts. Data collection, collation and analysis, together with pilot evaluation were conducted from January to June 2015.

### Program and pilot design

Based upon the DOTS strategy, national and local TB programs, the integrated control program aimed to improve case detection by two pathways: diverse outreach TB educational activities, and household screening targeting on TB suspects and high-risk groups (Table 1).

**Table 1:** Overview of pilot interventions to improve case detection in underdeveloped multi-ethnic regions in China, 2014-2015

Objectives	Interventions	Contents
Improve case detection by outreach TB education	1. Development of tailor-made TB educational materials	The tailor-made TB information sheet which contained TB knowledge and policy of early detection and insurance were developed in mandarin. Given a large proportion of the Uygur in ethnic minorities, it was accordingly translated by provincial TB experts, revised and finalized by discussion with professionals of Uygur language and Muslim.
	2. Health promotion to preach TB knowledge to the Muslims by Imams regularly in Masjids	In the Muslims, imam plays a key role in religion preach and promotion of knowledge, attitude and practice from public health perspective. In the pilot, the imams were organized and trained by TB experts. In Masjids, they read and explained TB knowledge and policy to the Muslims in regular preach for at least four times, distributed the TB information sheet, asked them to deliver the information to their family members and friends, as well as referred TB suspects for further diagnosis.
	3. Health promotion to educate students by school teachers and deliver TB knowledge to their family members	TB education for students is acknowledged as another effective approach not only benefits the students in their whole lives but also benefits their family members. In the pilot, the teachers in elementary and junior high schools were organized and trained by TB experts. All students were accordingly educated by the teachers, promoted to read the TB information sheet to their family members and to feed back the completion performance by receipts (See in Additional file 4). TB suspects in the students were referred for further diagnosis by the school teachers.
	4. Health promotion to each household by village doctors	In village communities, the outreach TB education in each household was conducted along with screening activities. All the residents, regardless of age and ethnics, were educated by trained village doctors in investigation teams using the TB information sheet. Each investigation team also included village coordinator and township doctor. For those who were not at home during the household education, their family members were promoted to conduct an education on behalf of the investigation teams.
Improve case detection by household screening	5. Household screening of people younger than 65 years old by suspicious pulmonary TB symptoms	According to the household registration, eligible resident population was investigated for symptoms by the investigation teams. For those who were not at home during the household screening, the symptoms investigations were conducted by mobile phone or a second investigation. The symptomatic persons were promoted to TB hospital for chest X-ray and sputum smear microscopy. The defaulters were regularly followed up by continuous investigation and health promotion.
	6. Household screening of older people by promoting to chest X-ray examination	All older people were promoted for free chest X-ray in the township health center by the investigation teams along with symptoms screening. Assistance from their family members or a second investigation was adopted for older people who were not at home during the screening. The defaulters were regularly followed up by continuous investigation and health promotion. Those identified with abnormal symptoms and/or chest X-ray results were referred to TB hospital for sputum smear microscopy. Chest X-ray test result within 1 year can be taken as reference.

The TB information sheet in both Chinese and Uygur language was developed and adopted in outreach education (Additional file 2). It comprised seven pieces of key information: (1) Pulmonary TB is a chronic respiratory infectious disease, transmitted by the droplet nuclei of patients when they cough, sneeze or speak; (2) Cough or expectoration for over 2 weeks and sputum with blood should be suspicious of pulmonary TB; (3) Those who present symptoms should attend TB hospital as quickly as possible for diagnosis and treatment; (4) Chest X-ray and sputum smear microscopy are provided free of charge for TB diagnosis; (5) The reimbursement of TB treatment is 100% for outpatient and 90% for inpatient by the new rural cooperative medical scheme; (6) The older people aged 65 years and over have high risk for TB. Free examinations are provided in the township health centres and TB hospital for early detection and treatment; (7) The large majority of TB patients can be cured by a good adherence to standardized treatment.

The household screening in the village communities targeted all eligible resident population: local resident population (excluded those who left for more than 6 months) and internal migrant population who lived in the pilot site for more than half a year. The older people in the pilot were particularly referred to those who were born before 31 December 1949. The symptoms in the household screening were defined as cough and expectoration more than 2 weeks, haemoptysis or sputum with blood. TB patients were provided with the standardized TB treatment according to the national guideline.

### Methods of pilot evaluation

We evaluated the pilot performance and effectiveness in both quantitative and qualitative ways. The data of operational results in outreach TB education and household screening were collected from work records in the field implementation. The work records were required by the implementation rules and included the record sheets for the educational activities, population verification, recruitment and screening in each household, as well as referral, tracing and diagnosis results in each TB suspect and elderly person. The number of TB suspects consulting doctors and TB patients detected before and after the pilot interventions were collected from the electronic TB Information Management System [18]. We also investigated 120 TB patients registered in Yining to examine their accessibility to the TB hospital. Semi-structured interview surveys were conducted to summarize the experiences, challenges, limitations and recommendations individually from 11 pilot managers and working staff. Ten older people who did not participate in the household screening were interviewed for the reason of absence. The outline of interview survey can be seen in Additional file 3.

### Operational results

Each trained imam in 22 Masjids promoted TB education in daily preaches. TB educational sessions were totally conducted in 88 times of preaches, covering 20,440 person-times for the Muslims (232 person-times in each preach on average). The trained teachers promoted TB education to 1944 students in 74 classes from five elementary and two junior high schools. The students also contributed to educating 6929 persons of their family members, where the feedback rate (proportion of those who fed back the completion performance by receipts among total family members) was 99% (6929/7020).

In the village communities, 13,073 residents participated in household education and screening, with a response rate of 93% (13,073/14057). Among them, the median age was 30 (interquartile range: 15 – 46) years old; 12,292 (94%) were aged under 65 years and 781 (6%) were aged 65 years and over; male accounted for around 53% in total, younger and older people; more than 99% (13,064/13073) were local resident population.

In people aged under 65 years, 93 cases were identified with symptoms out of 12,292 investigated participants, and 96% (89/93) were exanimated in TB hospital. By chest X-ray examinations, 14 cases presented active TB lesion and two cases presented inactive TB lesion. Six cases were finally diagnosed together with sputum smear microscopy. The estimated prevalence was 49/100000, and the number needed to screen to detect one case of active TB patient (NNS) was 2049 (12,292/6).

Among older people, 89% (692/781) were promoted and examined by chest X-ray examination in the township health centre. Among 76 cases presenting abnormal chest X-ray for TB, 39 were having active TB lesion. Ten patients were finally diagnosed together with sputum smear microscopy, while only two of them had symptoms. The estimated prevalence was 1445/100000, and the NNS was 78 (781/10).

**Fig. 2:**
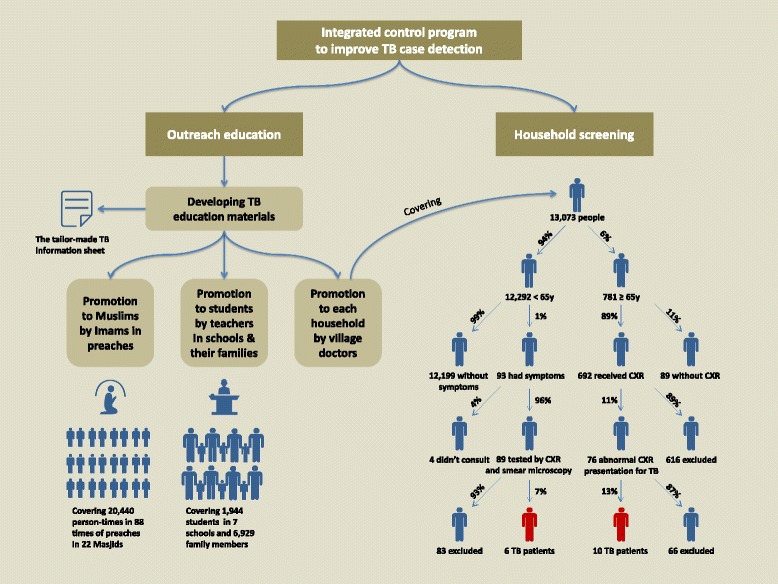
The conceptual framework and operational results of pilot interventions to improve case detection in underdeveloped multi-ethnic regions in China, 2014 – 2015 (TB: tuberculosis, 65 y: 65 years old, CXR: chest X-ray)

The operational results of pilot interventions are shown in Fig. 2.

### Experience learned

In the pilot implementation, supportive healthcare system, multi-sectoral cooperation and multi-channel financing mechanism were key experiences learned from the interview to enable the program accessible, available, affordable and eventually sustainable.

In the record of 120 TB patients registered in Yining, the average distance from the villages where they lived to TB hospital was 38 km (ranged from 2 to 110 km), which would take on average 67 min (ranged from 10 min to 3 h) for one way trip. In such remote areas, primary healthcare services are essential for early detection. In the pilot, township and village doctors played a crucial role in identifying TB suspects by household education and screening. By designating high-quality clinical services in TB hospital, CDC was specialized in program management, such as developing education approaches, training pilot members, and following up TB suspects and defaulters.

Given the complex social determinants, efforts from health department alone are inadequate. In the pilot, administrative staffs like village coordinators were designated as leaders in the investigation teams, as they were more familiar with household status and trusted for a walk-in investigation. The engagements of imams and teachers were successful attempts in TB education programs, and highly recommended from public health perspective. The role and leadership of governments in coordinating related departments should not be ignored.

Financing for public health interventions is usually more difficult in underdeveloped regions. Since the 1990s, international projects accounted for a large proportion of total TB budget and contributed significantly to the scale-up of DOTS strategy in Xinjiang. Along with the end of external donations, it is the government’s accountability to guarantee adequate resources within a sustainable system. So in the pilot design, the majority of activities were commissioned by local governments through different channels. Incentives were given to the imams, school teachers, household investigation teams and older people. All screening interventions including the chest X-ray, sputum smear microscopy and the transportation from village to township health center for the elderly screening were provided free of charge. Other clinical services related to TB (including differential diagnosis, drugs and follow-up treatment) were covered by health insurance with no less than 90% of reimbursement rate.

### Pilot effectiveness and evaluation

We evaluated the pilot effectiveness by comparing detection performance before and after the interventions. The number of TB suspects consulting village doctors and referred to TB hospital was 47 in the first quarter in 2015, which was two times higher than that of 23 in corresponding period of 2014. Among the 47 cases, eight consulted doctors ascribed to the TB knowledge learned from imams’ preaches, ten from students promotion, and eight from household education. This number roughly accounted for the incremental size. From November 2014 to April 2015, 34 new TB patients were notified compared to 27 cases in previous corresponding period.

From the evaluated outcome, the case detection appears to be effectively improved. Among the TB patients detected, 47% (16/34) were promoted for early detection by household screening and 26% (7/27) were additionally diagnosed by integrated programs. Given the estimation that around 66% of TB patients were undetected in Xinjiang (even higher in Uygur) [4], however, question on whether we can further expand the case detection potential remains to be answered.

In the interview, most of the managers and working staff agreed the fact that more TB cases can be detected by continuous and expanded programs, though it was hard to reach a consensus of the increasing extent and whether it is worthwhile. The key findings in the household screening presented a consistently high TB prevalence in older people, but a relatively lower rate in people aged under 65 years compared to the Xinjiang survey [4]. One reason might be the low diagnostic yield of symptoms screening. In the national survey, nearly half TB patients did not present cough; the sensitivity of cough more than 2 weeks in screening was less than 45% [19]. In our pilot, therefore, older people were designated for chest X-ray regardless of symptoms given their higher TB burden and atypical symptoms [20]. The fact that two out of ten elderly TB patients had symptoms proved lower diagnostic value of symptoms screening alone. Conversely, the symptoms screening in younger people might lead to at least half patients undetected. Compared to elderly TB patients, younger patients usually presents acute coughs [19]. Current threshold of cough more than 2 weeks might also be responsible for a lower patient diagnosis rate.

More aggressive screening strategy in younger people may improve case detection. However, indiscriminate mass screening should be very careful especially in such underdeveloped regions. In addition to the screening performance and diagnostic yield, cost-effectiveness, feasibility and acceptability should also be taken into account. The NNS can be a rough indicator of cost-effectiveness and effort. NNS to identify one true case of TB is theoretically identical to the inverse of the prevalence of detectable TB. According to one systematic review from WHO, the NNS in younger people in this case study was higher than that in most groups reported in intermediate and high TB incidence countries [16]. It may imply the cost-ineffectiveness assuming the costs and the yields are the same across risk groups. Although the NNS can be decreased by adopting more accurate diagnostic tools, the cost may significantly increase. Moreover, the feasibility and acceptability could become problematic with potential challenges.

### Potential challenges

Despite the progress made, conducting household screening in such context is still challenging. According to the interview, heavy workload and inadequate healthcare workers in remote rural areas were mainly concerned for sustainable development. The screening consumed plenty of time and human resource in verifying household members, obtaining the acceptance, organizing field work and activities to ensure a high response rate. In addition to the limited number of healthcare workers, personnel expertise and performance could be another issue. The relatively lower educational level and professional competence in some village doctors might affect their understanding of pilot interventions, the effectiveness of training and participations in TB education.

More attentions should also be paid to the acceptability of household screening. In the pilot, 11% of older people did not consult for chest X-ray in township health centre regardless of free clinical services, transportation and incentives. By interviewing ten of them, we found they were all covered by primary health insurance and willing to consult for clinical services when they had illness. They all declared that free screening for TB was helpful and they were educated and mobilized for screening two to four times. However, the awareness of TB symptoms and policy in some of them were obscure according to their responses. Regarding the reasons of absence, four of them were due to physical inconvenience caused by chronic diseases at the time of screening, while others declared “good health status and no history of critical illness”, “no symptoms and no need for screening”, “personal affairs” and “already participating in health examination recently”. By further promoting TB knowledge, especially the risk of transmission to family members, half of them would like to participate in future screening in feasible ways, while others were still reluctant given their good health status.

### Limitations of pilot design

Considering the additional workload and difficulties in application, we did not examine the training effectiveness for working staff and the awareness of TB knowledge in diverse participants before and after the pilot interventions. Targeted evaluation would provide more direct and practical evidence for improving the program design. According to the interview, it was regarded that the majority of training and educational activities were conducted in a paternalistic way. More intensive training and educational approaches, such as developing visualized materials, multiple channels to integrate TB knowledge into religious stories, role-playing or interactive games among teachers, students and family members were recommended. Regarding the household screening, impacts of early detection on reducing diagnosis delay, transmission and mortality, as well as costs and cost-effectiveness were beyond the scope of this case study. Further epidemiological and economic evaluations were warranted.

### Implications and recommendations

Given the high disease burden and suboptimal case detection performance, more TB patients were expected to be detected in China underdeveloped multi-ethnic regions. However, how and to what extent the case detection can be improved had not been well examined. Although outreach education has been proved as an effective approach for improving access to patient care [21], and the systematic screening for TB had been studied in aspects of methodology, algorithms and different high-risk groups, the direct evidence remains weak for multi-ethnic regions and older people [16, 20]. This case study demonstrated a target pilot of integrated control program to improve TB case detection, which can inform the further TB control programs in similar areas.

All the managers and working staff in the interview acknowledged the necessity and improved effectiveness of interventions. They recommended sustainable development and scale-up of the program in line with following issues:

- Great importance should be attached to the supportive healthcare system, multi-sectoral cooperation and multi-channel financing mechanism;
- The human resources especially in primary healthcare needs further improvement;
- More intensive TB education approaches for specific groups and training approaches for healthcare workers are recommended to be developed and evaluated;
- A high index of TB suspicion, such as lowering the symptoms threshold in people aged under 65 years, and increasing the awareness of atypical clinical presentation in older people, would increase the yield of case detection;
- Indiscriminate mass screening should be very careful. Social-economic situation, healthcare system, demographic and epidemiologic characteristics, the performance and cost-effectiveness of approaches should be taken into account;
- Given the high disease burden, interventions in older people are recommended to be prioritized towards End TB goals [20]. To increase the feasibility, TB screening in older people could be integrated into primary public health services programs, such as annual health examination or screening program for other chronic diseases;
- The impacts of interventions on reducing disease burden, and the cost-effectiveness, feasibility and acceptability issues warrant further research and evaluation in each specific context.

## Conclusions

In underdeveloped multi-ethnic regions with high TB burden, the integrated control program to improve case detection is necessary and can be feasible and effective under supportive healthcare system, collaborative cooperation and affordable financing mechanism. The diverse approaches of outreach education and household screening effectively improved case detection compared to the previous performance. More intensive educational approaches, enhanced training to improve personnel capacity, a high index of TB suspicion, and prioritization of older people in screening are recommended. In order to sustain and scale up the program, the impacts, cost-effectiveness, feasibility and acceptability of interventions warrant further research and evaluation in each specific context.

## Additional files

Additional file 1: Multilingual abstracts in the five official working languages of the United Nations. (PDF 698 kb)

Additional file 2: The Chinese and Uygur version of the TB information sheet. (DOCX 273 kb)

Additional file 3: The outline of interview survey. (DOCX 901 kb)

Additional file 4: The Chinese and Uygur version of receipt for completing outreach TB education in family members (DOCX 23 kb)

## Abbreviations

CDC: Centre for disease control and prevention
DOTS: Directly observed treatment, short-course
GDP: Gross domestic product
NNS: Number needed to screen to detect one case of active TB patient
TB: Tuberculosis
WHO: World Health Organization
