# Supplementary material for: Improving tuberculosis case detection in underdeveloped multi-ethnic regions with high disease burden: a case study of integrated control program in China
Source: Infect Dis Poverty. 2017 Nov 29;6:151. doi: 10.1186/s40249-017-0365-4 (PMC5706405; doi:10.1186/s40249-017-0365-4)
Supplement: Supplementary file 1 — Multilingual abstracts in the five official working languages of the United Nations. (PDF 698 kb) [file 40249_2017_365_MOESM1_ESM.pdf]

Translation of the abstract into the five official working languages of the United Nations

تحسين الكشف عن حالات السل في المناطق المتخلفة متعددة الأعراق ذات العبء المرتفع للأمراض: دراسة حالة لبرنامج المراقبة المتكامل في الصين

جون لي، وشياو - تشيو ليو، وشي ون جيانغ، وشيويه لى، وفاي يو، ويان وانغ، ويونغ بينغ، وشياو مينغ قو، ويان ني سون، وهوى تشانغ و لى شيا وانغ

ملخص

لمحة عامة: في المناطق المتخلفة متعددة الأعراق في الصين، يزيد العبء المرتفع من السل وعدم المساواة الإقليمية في الحصول على خدمات الرعاية الصحية من التحدي المتمثل في تحقيق أهداف القضاء على السل. ومن بين جميع المقاطعات، سجلت أعلى نسبة للسل في شينجيانغ، حيث عانت الأقليات العرقية وكبار السن أكثر من غيرهم. غير أن الاستراتيجية الحالية لتقصي الحقائق غير كافية بالنظر إلى المحددات الاجتماعية المعقدة ومعدلات الكشف عن الحالات دون المستوى الأمثل. وهكذا، وضعنا برنامجا متكاملًا لمكافحة السل من أجل تحسين كشف الحالات، ونفذنا تجربة تجريبيةفي شينجيانغ في الفترة من 2014 إلى 2015. في دراسة الحالة هذه، قمنا بتلخيص الأنشطة والنتائج الرئيسية. كما شاركنا التجارب والتحديات التي تواجه تنفيذ التدخلات وقدمنا توصيات لإبلاغ برنامج مكافحة السل في المستقبل.

عرض حالة: تم تنفيذ التدخلات التجريبية في بلدة مختارة في يينينغ استنادًا إلى برامج مكافحة السل المحلية. من خلال تطبيق مواد تعليمية مصممة خصيصًا، تم الاضطلاع بأنشطة تثقيفية في مجال التوعية بالسل بطرق مختلفة. وفي 22 مسجد، قام الأئمة المدربون بترويج التوعية الخاصة بمرض السل للمسلمين، وغطى 2040 شخصًا في 88 جلسة تم تنظيمها في الخطب. وفي سبع مدارس، رفع المعلمون وعي 1 944 طالبًا وأسهموا في تثقيف 6 929 من أفراد الأسر. وفي المجتمعات القروية، شارك13 073 من السكان في تعليم الأسر المعيشية وفحصها. ومن بين هؤلاء، تم التحقيق في 12 292 شخصًا دون الخامسة والستين من العمر لأعراض مرض السل الرئوي المشبوهة، حيث تم تشخيص ستة مرضى بالسل من أصل 89 مشتبهًا فيهم؛ تم حشد 781 من كبار السن للفحص مباشرة عن طريق الصدر بالأشعة السينية، حيث تم تشخيص 10 مرضى من أصل 692 مشاركًا. وكان نظام الرعاية الصحية الداعمة والتعاون المتعدد القطاعات وآلية التمويل متعددة القنوات هم التجارب الناجحة للتنفيذ. وقد ثبت أن التدخلات أكثر فعالية من الأداء السابق: فقد زاد عدد المشتبه بهم الذين استشاروا الأطباء والمرضى الذين تم الكشف عنهم بنسبة 50٪ و 26٪ على التوالي. وينبغي أن تؤخذ في الحسبان التحديات والآثار والتوصيات المحتملة لزيادة تحسين البرامج.

الاستنتاجات: في المناطق المتعددة الأعراق المتخلفة ذات العبء المرتفع للسل، من الضروري تحسين الكشف عن الحالات، ويمكن أن تكون التدخلات ممكنة وفعالة في إطار نظام داعم. ويوصى باتباع مناهج تعليمية وتدريبية أكثر كثافة، ومؤشر مرتفع للاشتباه في السل وتحديد الأولويات لكبار السن في الفحص. ومن أجل الحفاظ على البرنامج وتوسيع نطاقه، فإن آثار التدخلات وفعاليتها من حيث التكلفة وجدواها وقبولها تستدعي مزيدًا من البحث والتقييم في كل سياق محدد.

Translated from English version into Arabic by Eman Shahen, through

TRANSLATORS WITHOUT BORDERS

在欠发达和多民族的结核病高负担地区提高患者发现水平：来自中国综合性防控项目的案例研究

Jun Li, Xiao-Qiu Liu, Shi-Wen Jiang, Xue Li, Fei Yu, Yan Wang, Yong Peng, Xiao-Ming Gu, Yan-Ni Sun, Hui Zhang and Li-Xia Wang

摘要

引言：在中国欠发达和多民族地区，结核病高负担和医疗卫生服务地区间分布不均是实现消除结核病目标的主要挑战。新疆结核病疫情在所有省份中最高，其中少数民族和老年人的疾病负担较重。考虑到复杂的社会影响因素和较低的患者发现率，新疆结核病发现策略亟待完善。因此，我们制定了提高患者发现水平的综合性结核病防控项目，并于 2014-2015 年在新疆开展了试点研究。在此案例分析中，我们总结了项目的主要措施和试点结果，分享了实施过程中取得的经验和面临的挑战，并为进一步完善防控项目提出建议。

案例分析：基于当地实际和现有结核病防控项目，我们选取了新疆伊宁县的一个乡镇开展试点干预措施。通过开发和使用针对性的结核病健康教育材料，项目扩展了不同方式的主动干预措施。在 22 个清真寺，接受培训的阿訇将结核病健康教育纳入常规宗教活动，在 88 场对穆斯林居民的宣讲中覆盖了 20 440 人次。在 7 所学校，培训后的教师对 1 944 名学生进行健康教育，并由学生完成了对 6 929 位家属的宣教。在乡村，13 073 位常住居民参加了入户健康教育和筛查。我们对其中的 12 292 位 65 岁以下居民开展了肺结核可疑症状筛查，89 例结核病可疑者中有 6 例确诊为结核病；对 781 位老年人提供了免费的胸片筛查，参加筛查的 692 例中确诊 10 例。支持的卫生服务体系、多部门合作和多渠道筹资机制是试点实施成功的主要经验。与同期相比，项目实施有效促进了患者发现水平：就诊的结核病可疑者数和发现的患者数分别提高了 50%和 26%。同时，此次试点总结的问题和建议为下一步项目完善和扩展提供借鉴。

结论：在中国欠发达和多民族的结核病高负担地区，防控项目对提高患者发现水平意义显著。基于支持的防控体系，此案例研究表明综合性干预措施可行且有效。研究建议进一步开发健康教育和培训方式，强化结核病可疑者的发现，以及将老年人作为筛查的重点人群。为扩展和可持续发展项目措施，还应针对不同地区情况进一步开展流行病学影响、成本效果、可行性和可接受度的研究和评价。

Translated from English version into Chinese by Jun Li

Améliorer la détection des cas de tuberculose dans les régions multiethniques sous-développées où le taux de morbidité de la maladie est très élevé: une étude de cas dans le cadre du programme de contrôle intégré en Chine

Jun Li, Xiao-Qiu Liu, Shi-Wen Jiang, Xue Li, Fei Yu, Yan Wang, Yong Peng, Xiao-Ming Gu, Yan-Ni Sun, Hui Zhang et Li-Xia Wang

Résumé

Contexte: Dans les régions multi-ethniques sous-développées de Chine, le taux de prévalence élevé de la tuberculose (TB) et l'inégalité régionale en matière d'accès aux services de santé augmentent les difficultés d'atteindre les objectifs pour enrayer la tuberculose. Parmi toutes les provinces, celle du Xinjiang s'est révélée être la plus sévèrement touchée par la tuberculose : les minorités ethniques et les personnes âgées sont ceux qui en ont le plus souffert. Cependant, la stratégie actuelle de recherche de cas est inappropriée compte tenu des déterminants sociaux

complexes et des taux de détection de cas sous-optimaux. Nous avons alors élaboré un programme intégré de contrôle de la tuberculose pour améliorer la détection des cas et avons lancé un projet pilote au Xinjiang de 2014 à 2015. Dans cette étude de cas, nous avons résumé les activités et les principales constatations. Nous avons également partagé les expériences et les difficultés de la mise en œuvre des interventions et avons formulé des recommandations qui enrichiront le programme de lutte contre la tuberculose dans le futur.

Présentation de cas: Les interventions pilotes ont été mises en œuvre dans une ville choisie, à Yining, sur la base de programmes locaux de lutte contre la tuberculose. En appliquant Matériels de formation conçus sur mesure**,** les activités d'action éducative de la tuberculose ont été menées de diverses façons. Dans 22 Masjids, les imams ayant reçu une formation ont encouragé les musulmans à s'informer de la tuberculose, couvrant 20 440 personnes-temps dans 88 lieux de prêche donnés. Dans sept écoles, 1 944 étudiants ont été sensibilisés par les professeurs et ont contribué à former 6 929 membres de leur famille. Dans les communautés villageoises, 13 073 résidents ont participé à l'éducation des ménages et au dépistage. Parmi eux, 12 292 personnes âgées de moins de 65 ans ont fait l'objet d'une enquête aux fins de détecter tout symptôme suspect de la tuberculose pulmonaire : sur 89 cas suspects de tuberculose, six cas de tuberculose ont été diagnostiqués ; 781 personnes âgées ont été rassemblées et ont bénéficié d'une radiographie pulmonaire pour un dépistage immédiat : sur 692 participants, 10 cas de tuberculose ont été diagnostiqués. Le système de soutien en matière de santé, la coopération multisectorielle et les dispositifs de financement à multi-canaux ont été les expériences réussies de la mise en œuvre. Les interventions se sont révélées plus efficaces que les résultats précédents: le nombre de patients suspectés d'être atteints de la tuberculose consultant des médecins et les cas détectés ont augmenté de 50% et 26% respectivement. Les difficultés éventuelles, les implications et les recommandations devraient être prises en considération pour améliorer davantage le programme**.**

Conclusions: Dans les régions multiethniques sous-développées les plus affectées par la tuberculose, améliorer le détection des cas est nécessaire et les interventions peuvent être réalisables et efficaces dans un système de soutien. Des approches en matière d'éducation et de formation plus intensives, un indice plus élevé de la suspicion de la tuberculose ainsi que la priorisation des personnes âgées dans le dépistage sont recommandés. Pour soutenir et intensifier le programme, les impacts, la rentabilité, la faisabilité et l'acceptabilité des interventions justifient une recherche et une évaluation supplémentaires dans chaque contexte spécifique.

Translated from English version into French by veromarie, through

TRANSLATORS
WITHOUT BORDERS

Повышение эффективности выявления случаев туберкулеза в слаборазвитых многонациональных регионах с повышенным уровнем распространенности заболевания: ситуационное исследование комплексной программы по борьбе с туберкулезом в Китае

Джун Ли, Сяо-Цю Лю, Ши-Вэнь Цзян, Сюэ Ли, Фэй Юй, Янь Ван, Юн Пэн, Сяо-Мин Гу, Янь-Ни Сун, Хуэй Чжан и Ли-Ся Ван

Аннотация

Контекст исследования: В слаборазвитых многонациональных регионах Китая высокий риск заболевания туберкулезом (ТБ) и региональное неравенство в доступе к услугам здравоохранения осложняют достижение цели "Положить конец ТБ". Среди всех провинций наиболее высокий уровень заболеваемости туберкулезом отмечается в Синьцзяне, где больше всего пострадали этнические меньшинства и пожилые люди. Однако нынешняя стратегия выявления случаев заболевания неэффективна, учитывая сложные социальные детерминанты и показатели выявления субоптимальных случаев. Таким образом, мы разработали комплексную программу борьбы с ТБ для повышения эффективности выявления случаев заболевания и провели пилотный эксперимент в Синьцзяне с 2014 по 2015 год. В данном ситуационном исследовании мы обобщили действия и основные выводы. Мы также поделились опытом внедрения мероприятий и предоставили рекомендации по информированию населения о программе борьбы с туберкулезом в будущем.

Описание ситуации: Экспериментальные вмешательства проводились в выбранном городе Инине на основе местных программ борьбы с туберкулезом. Применяя индивидуальные учебные материалы, разными способами проводилась информационно-просветительская деятельность в области борьбы с ТБ. В 22 Масджидах обученные имамы информировали мусульман об опасностях туберкулеза, охватив 20 440 человек за 88 прочитанных проповедей. В семи школах 1 944 учащихся получили информацию о туберкулезе от своих учителей и способствовали информированию 6 929 членов семьи. В сельских общинах в обучении и обследовании приняли участие 13 073 жителя. Среди них 12 292 человека в возрасте до 65 лет прошли обследование на наличие подозрительных симптомов туберкулеза легких, в результате у шести из 89 пациентов с подозрением на заболевание был диагностирован туберкулез. 781 пожилой человек был отправлен на флюорографическое обследование, в результате у 10 из 692 участников был диагностирован туберкулез. Успешным опытом реализации вмешательства является благоприятная система здравоохранения, многосекторальное сотрудничество и механизм многоканального финансирования. Было доказано, что вмешательство повысило эффективность борьбы с туберкулезом: количество обращений пациентов с подозрением на туберкулез к врачам и количество выявленных случаев туберкулеза увеличилось на 50% и 26% соответственно. Для дальнейшего усовершенствования программы необходимо учитывать потенциальные трудности, последствия и рекоммендации.

Выводы: В слаборазвитых многонациональных регионах с высоким уровнем заболеваемости туберкулезом необходимо повышать уровень выявления случаев заболевания; вмешательство может послужить осуществимым и эффективным механизмом в рамках благоприятной системы. Рекомендуется применение более эффективных образовательных и обучающих подходов, высокого показателя подозрения на туберкулез и приоритетности пожилых людей при обследовании. Воздействие, экономическая эффективность, осуществимость и приемлемость вмешательств требуют дальнейших исследований и оценки в каждом конкретном контексте для дальнейшего поддержания и расширения программы.

Translated from English version into Russian by Karina1207, through

TRANSLATORS
WITHOUT BORDERS

Mejorar la detección de casos de tuberculosis en regiones subdesarrolladas multiétnicas con alta carga de morbilidad: Un estudio de caso del programa de control integrado en China

Jun Li, Xiao-Qiu Liu, Shi-Wen Jiang, Li Xue, Yu Fei, Yan Wang, Yong Peng, Gu Xiao-Ming, Yan-Ni Sun, Hui Zhang y Li-Xia Wang

Resumen

Contexto: En las regiones subdesarrolladas multiétnicas de China, la alta carga de tuberculosis (TB) y la desigualdad regional en el acceso al servicio de salud aumentan el reto de alcanzar los objetivos finales de la TB. Entre todas las provincias, la mayor carga de TB se registra en Xinjiang, donde las minorías étnicas y las personas mayores han sufrido más. Sin embargo, la actual estrategia de detección de casos es inadecuada dada la complejidad de los determinantes sociales y las insuficientes tasas de detección de casos. Así, desarrollamos un programa integrado de control de la TB para mejorar la detección de casos y realizamos un programa piloto en Xinjiang de 2014 a 2015. En este estudio monográfico, resumimos las actividades y los hallazgos clave. También compartimos las experiencias y desafíos de implantar las intervenciones y brindamos recomendaciones para informar al programa de control de TB en el futuro.

Presentación del caso: Las intervenciones piloto se implantaron en una ciudad seleccionada de Yining basada en programas locales de control de la TB. Mediante la aplicación de materiales educativos a medida, las actividades educativas de divulgación de la TB se realizaron de distintas formas. En 22 mezquitas, los imanes entrenados informaron sobre la TB a los musulmanes, llegando a 20 440 personas en 88 sesiones de predicación. En siete escuelas, 1 944 estudiantes fueron educados por los maestros y contribuyeron a educar a 6 929 miembros de sus familias. En las comunidades rurales, 13 073 residentes participaron en la educación del hogar y en el cribado. Entre ellos, 12 292 personas menores de 65 años fueron examinadas por síntomas sospechosos de TB pulmonar. Se diagnosticaron seis pacientes con TB de 89 sospechosos de tuberculosis; 781 personas mayores se movilizaron para la detección directa mediante radiografía de tórax y se diagnosticaron 10 casos de 692 participantes. El sistema de asistencia sanitaria solidario, la cooperación multisectorial y el mecanismo de financiación de múltiples canales fueron los éxitos de aplicación. Las intervenciones demostraron ser más eficaces que el anterior procedimiento: el número de sospechosos de TB que consultaron a un médico y los pacientes detectados aumentó en un 50% y 26%, respectivamente. Los posibles desafíos, implicaciones y recomendaciones deberían tenerse en cuenta para la mejora ulterior del programa.

Conclusiones: En regiones subdesarrolladas multiétnicas con alta carga de TB, es necesario mejorar la detección de casos y las intervenciones pueden ser factibles y eficaces dentro de un sistema de apoyo. Se recomiendan enfoques educativos y de formación más intensivos, un alto índice de sospecha de TB y priorizar a las personas mayores en el cribado. Mantener y ampliar el programa,

el impacto, la rentabilidad, la viabilidad y la aceptación de las intervenciones garantizan más investigación y evaluación en cada contexto específico.

Translated from English version into Spanish by Amparo Muñoz, through

TRANSLATORS
WITHOUT BORDERS
