# Supplementary material for: Improving tuberculosis case detection in underdeveloped multi-ethnic regions with high disease burden: a case study of integrated control program in China
Source: Infect Dis Poverty. 2017 Nov 29;6:151. doi: 10.1186/s40249-017-0365-4 (PMC5706405; doi:10.1186/s40249-017-0365-4)

**Additional file 4**

The Chinese and Uygur version of receipt for

completing outreach TB education in family members


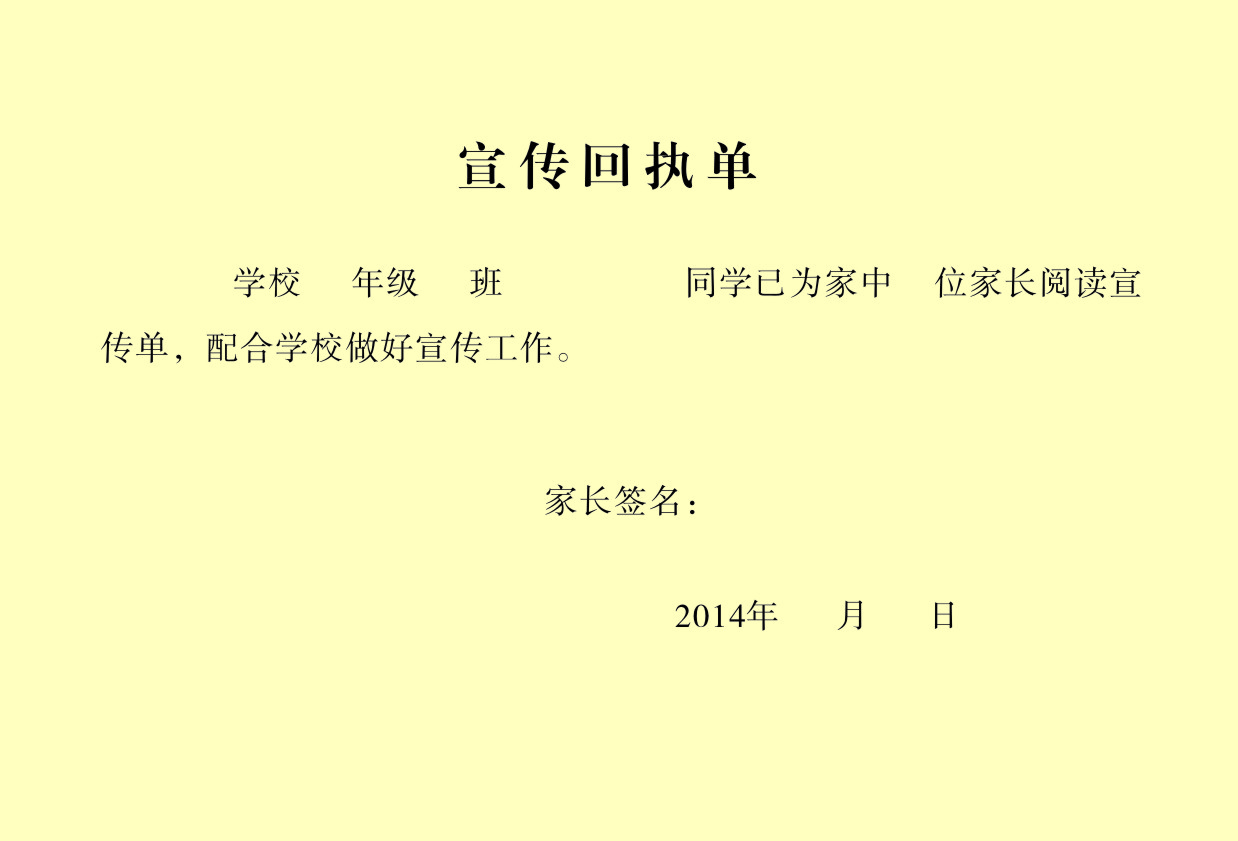


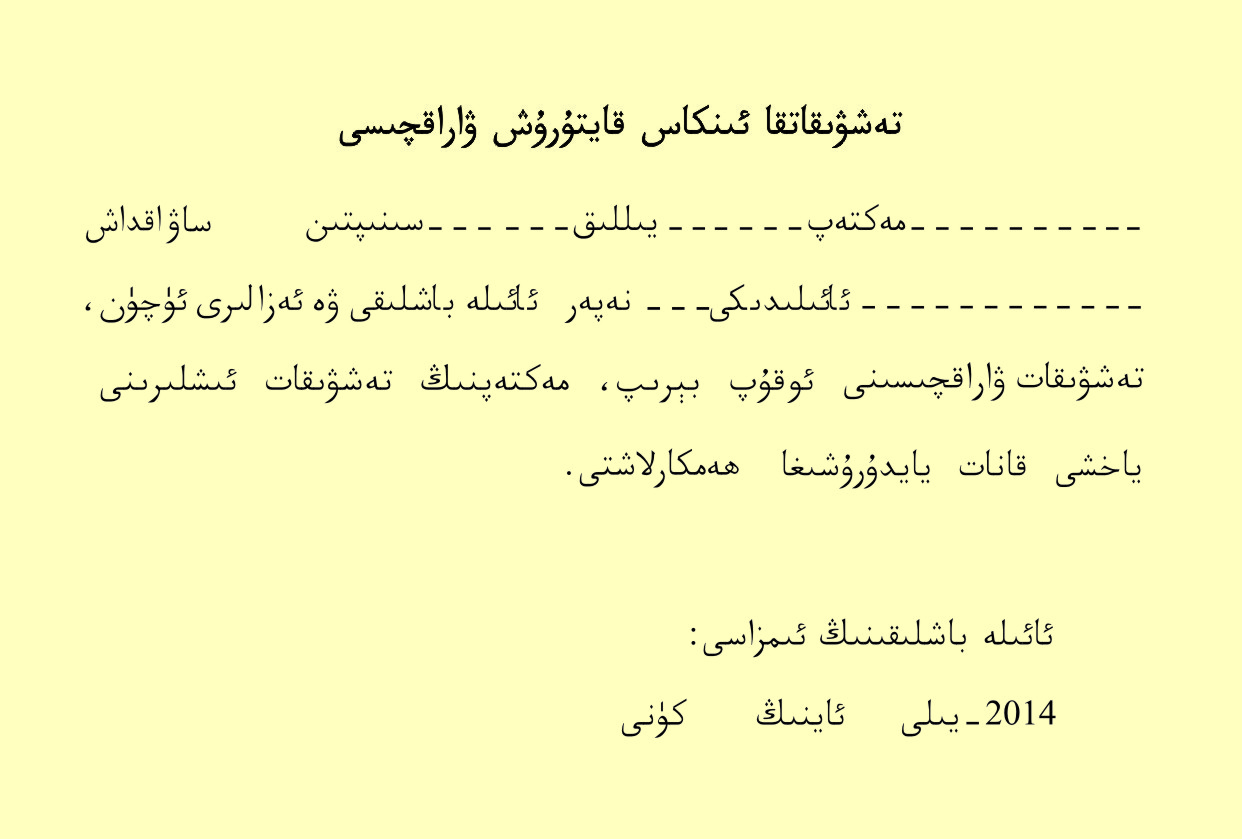

Supplement: Supplementary file 2 — The Chinese and Uygur version of the TB information sheet. (DOCX 273 kb) [file 40249_2017_365_MOESM4_ESM.docx]
