# Supplementary material for: Improving tuberculosis case detection in underdeveloped multi-ethnic regions with high disease burden: a case study of integrated control program in China
Source: Infect Dis Poverty. 2017 Nov 29;6:151. doi: 10.1186/s40249-017-0365-4 (PMC5706405; doi:10.1186/s40249-017-0365-4)

**Additional file 2**

The Chinese version of the TB information sheet


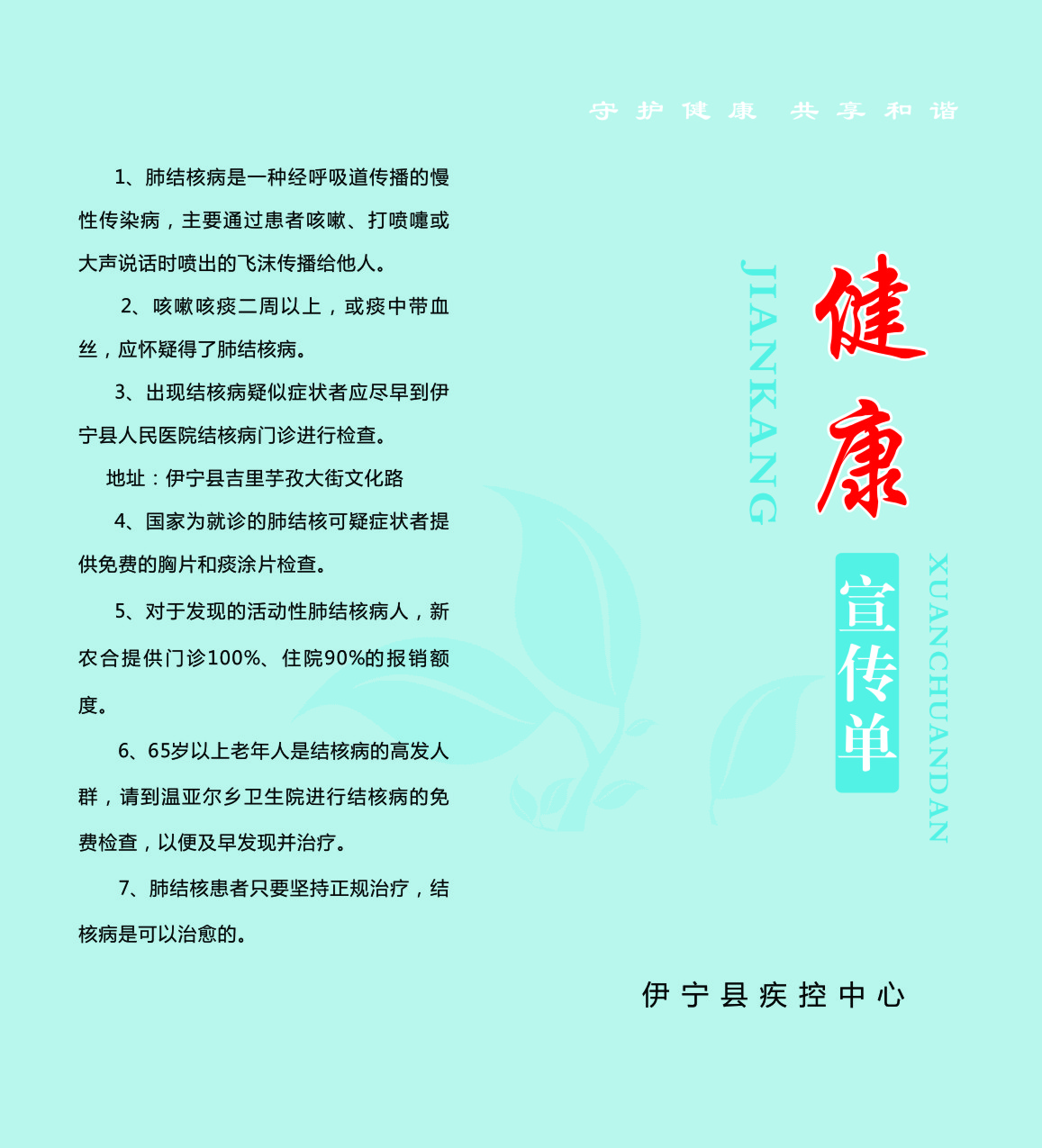


The Uygur version of the TB information sheet


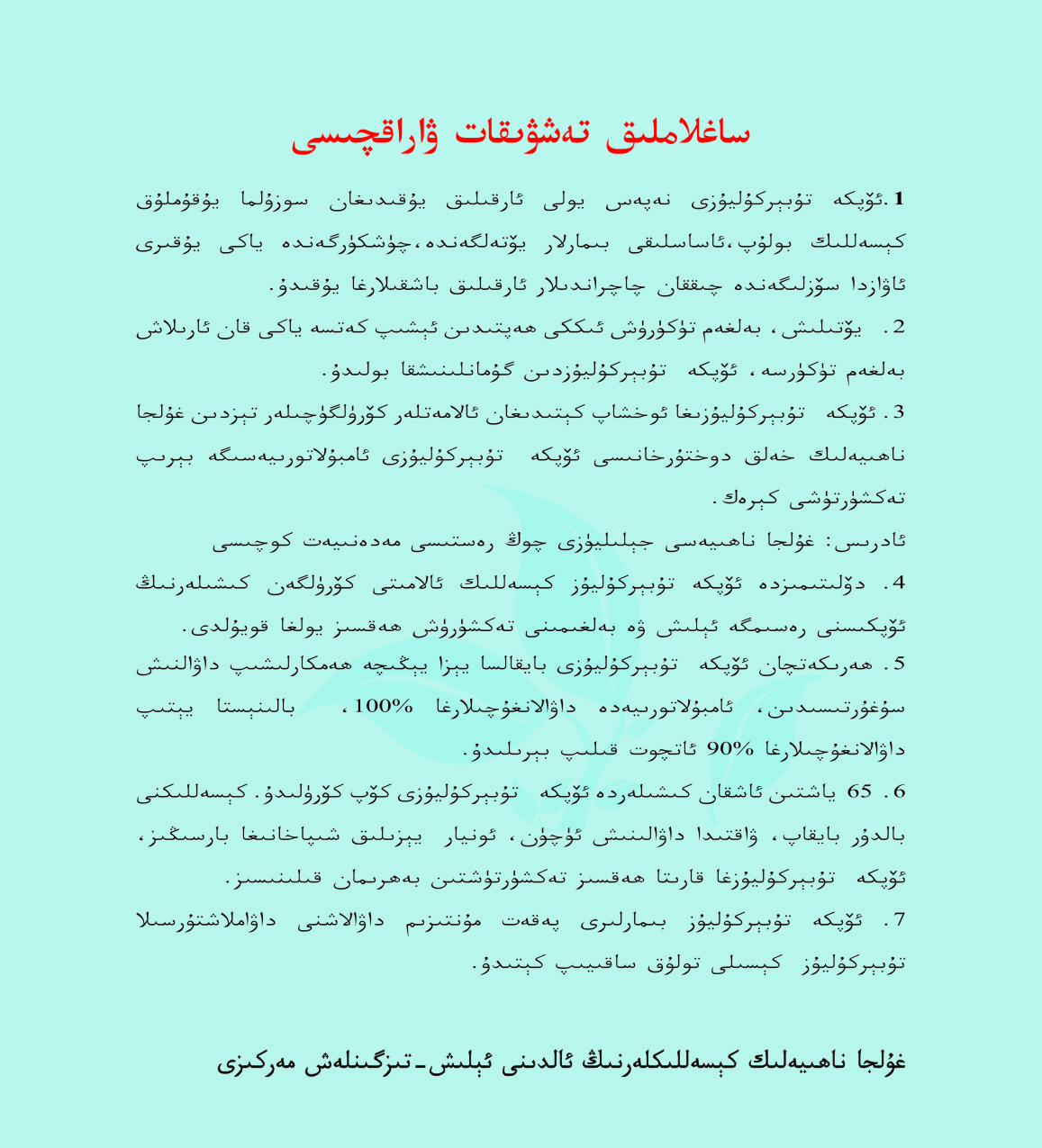

Supplement: Supplementary file 3 — The outline of interview survey. (DOCX 901 kb) [file 40249_2017_365_MOESM2_ESM.docx]
