# Supplementary material for: Improving tuberculosis case detection in underdeveloped multi-ethnic regions with high disease burden: a case study of integrated control program in China
Source: Infect Dis Poverty. 2017 Nov 29;6:151. doi: 10.1186/s40249-017-0365-4 (PMC5706405; doi:10.1186/s40249-017-0365-4)
Supplement: Supplementary file 4 — The Chinese and Uygur version of receipt for completing outreach TB education in family members (DOCX 23 kb) [file 40249_2017_365_MOESM3_ESM.docx]

**Additional file 3**

The outline of interview survey

1. Aim

To learn and summarize pilot experience, challenges, limitations and recommendations, and to provide evidence to improve integrated TB control program of early case detection in underdeveloped multi-ethnic regions with high disease burdens in China.

1. Interview objects
   1. Managers and working staff participated in the pilot

- Provincial level: one leader and one working stuff from Xinjiang CDC
- Prefecture level: one leader and one working stuff from Yili CDC
- County level: one leader and one working stuff from Yining CDC; one doctor from Yining TB hospital
- Township level: one leader and one working stuff from the township healthcare center
- Village level: two village doctors
  1. Ten older people who didn’t participate in the household screening

1. Interview outline
   1. Interview outline for managers and working staff

- What do you think are the key experiences learned in the pilot implementation?
- Currently, do you think the potential of case-finding in the pilot site can be further improved? Please specify your reasons.
- How would you evaluate the performance and effectiveness of following outreach TB education and household screening activities? What are the advantages and challenges? Do you think the interventions should be continuously conducted and why? What are your further suggestions and recommendations?
  - Health promotion to preach TB knowledge to the Muslims by Imams regularly in Masjids
  - Health promotion to educate students by school teachers and delivery TB knowledge to their family members
  - Health promotion to each household by village doctors
  - Household screening of people younger than 65 years old by suspicious pulmonary TB symptoms
  - Household screening of older people by promoting to chest X-ray examination
- How would you evaluate the program design, pilot organization and implementation? Please specify your reasons.
- What are your further suggestions and recommendations for the improvement and scale-up of program?
  1. Interview outline for older people absence of household screening
- How is your health state during the household screening? Have you got any type of serious diseases?
- Where would you initially consult for health service when you feel sick?
- How many family members in your household? What is the annual income? Have you participate in the health insurance program? If yes, what is the type?
- Did you know about the free household screening for TB in the older people couple of months ago? If yes, how did you learn it?
- Have you been promoted to free household screening for TB by village doctors? If yes, how many times they came to your house?
- Do you think the free household screening for TB is good for the older people? Please specify your reasons.
- We heard that you didn’t participate in the free household screening for TB, would you like to share your reasons with us?
- If there is another free household screening program for TB, would you consider participating in the future?
- What are your suggestions and recommendations for improving the program?
